# Sensory–Chemical Co-Dynamics in *Kadsura coccinea*: ROAV-Driven Prioritization of Cultivar-Specific Odorants and Mechanistic Validation via Molecular Docking

**DOI:** 10.3390/foods14213603

**Published:** 2025-10-23

**Authors:** Lin Wang, Ruiyin Zhang, Huilan Wu, Juan Xie, Qi Tang, Zhen Dong

**Affiliations:** 1 Shiyan Key Laboratory of Biological Resources and Eco-Environmental Protection, College of Chemical and Environmental Engineering, Hanjiang Normal University, Shiyan 442000, China; wl92606@126.com; 2 College of Horticulture, Hunan Agricultural University, Changsha 410128, China; 13548651231@163.com; 3 Chinese Medicinal Materials Breeding Innovation Centre of Yuelushan Laboratory, Changsha 410128, China; 4 College of Biosciences and Technology, Hunan Agricultural University, Changsha 410128, China; zry980610@163.com; 5 Hunan Province Key Laboratory of Traditional Chinese Veterinary Medicine, Hunan Agricultural University, Changsha 410128, China; 15608447717@163.com

**Keywords:** *Kadsura coccinea*, volatile organic compounds, cultivar differentiation, γ-dodecalactone, HS-SPME, GC-MS, olfactory receptor

## Abstract

This study deciphered the aroma differences in three *Kadsura coccinea* cultivars (F023, F054, F055) through integrated volatile-omics and sensory analysis. HS-SPME-GC-MS identified 49 volatiles dominated by sesquiterpenes (65.2–78.4%). ROAV prioritization revealed cultivar-specific drivers: γ-dodecalactone (ROAV = 73.0) defined F054’s fruity–floral character; humulene (ROAV = 100) and β-caryophyllene shaped F023’s woody–pungent profile; and β-pinene (ROAV = 100) characterized F055’s herbaceous freshness. Molecular docking confirmed high-affinity binding of γ-dodecalactone to OR2W1 (ΔG = −6.42 kcal/mol via ASN155 H-bonding). Sensory PCA explained 83.48% of the variance, segregating cultivars along distinct axes (F054: sweet-aromatic; F023: woody-spicy; F055: herbaceous-fresh). Joint PCA validated γ-dodecalactone–coconut milk spatial co-localization (θ < 10°) and β-caryophyllene–woody note correlations (r > 0.9), establishing γ-dodecalactone as a breeding biomarker for aroma-driven cultivar improvement.

## 1. Introduction

*Kadsura coccinea* (Lem.) A. C. Smith (KC), a rare woody liana of the family Schisandraceae, is primarily distributed in southern China with sparse populations documented in Vietnam. In traditional ethnic medicine systems of China, its roots and vines have been utilized to treat gastrointestinal inflammation, dysmenorrhea, and arthralgia [1,2,3], with lignans and triterpenoids identified as the principal bioactive constituents [4]. The mature fruit presents as an aggregate structure exhibiting pink to dark red coloration, characterized by a distinctive aroma described as a “sweet, apple-like fragrance with cooling undertones” [5]. While historically consumed primarily by indigenous communities within its native range, the fruit has garnered broader consumer interest due to its striking morphological appearance (colloquially termed “devil fruit”). The species demonstrates remarkable environmental resilience, thriving in acidic sandy loam soils with tolerance to thermal extremes (−15 °C to >40 °C). Minimal cultivation management is required beyond pollination and irrigation, facilitating the recent expansion of commercial cultivation. The edible pulp possesses a sweet–sour sensory profile and is nutritionally enriched with vitamins, carbohydrates, and essential amino acids [6], earning the designation “fruit of longevity and beauty” among Hmong people. Contemporary phytochemical analyses confirm high concentrations of polysaccharides and polyphenols in the fruit, correlating with significant antioxidant capacity [7,8,9].

As an emerging specialty fruit, research on KC fruits has predominantly focused on nutritional profiles and health-promoting properties. However, with escalating consumer demand for fruit products, olfactory characteristics have emerged as a primary driver of consumer preference alongside taste attributes [10,11]. Concurrently, aroma compounds serve as critical quality indicators in KC’s traditional medicinal applications, where more intense aromas correlate with enhanced therapeutic efficacy—a key parameter in ethnopharmacological evaluations. Volatile aroma compounds represent essential metabolites in fruits, comprising a diverse array of chemical classes such as esters, aldehydes, alcohols, terpenoids, and ketones. The distinctive flavour of fruit is largely governed by the precise composition and abundance of these volatile substances, which collectively contribute to the unique aromatic characteristics that differentiate various fruit cultivars [12,13]. A single ethnopharmacological analysis identified 85 compounds in hydro distilled essential oils, revealing dominant sesquiterpenes and oxygenated sesquiterpenoids (e.g., β-caryophyllene, γ-amorphene) in peel/pulp, while seeds contained primarily aliphatic fatty acids (e.g., n-hexadecanoic acid, linoleic acid) [14]. Hydro distillation of KC vegetative tissues highlightedα/β-pinene as the major constituents in roots, stems, and leaves [15]. However, these studies have focused on biological activities. Some key flavour volatiles exhibit significant divergence across cultivars and ripening stages [16,17]. A prior work on related omija fruits (e.g., *Schisandra chinensis*) identified α-pinene, α-terpinene, and (E)-β-ocimene as primary discriminators of inter-freezing treatments aroma differences [18]. However, no systematic analysis exists correlating volatile composition with sensory attributes across KC cultivars.

Traditional techniques for extracting volatile compounds, primarily steam distillation and solvent extraction, have been historically employed for the isolation of aroma substances [19,20]. However, these conventional approaches are hampered by several intrinsic limitations, including the thermal degradation or alteration of heat-sensitive compounds, substantial sample requirements leading to low efficiency, and a reliance on organic solvents that raise environmental concerns. In recent years, Headspace Solid-Phase Microextraction (HS-SPME) has gained widespread adoption for the extraction of fruit aroma compounds. Compared to conventional methods, HS-SPME offers significant advantages, including high sensitivity, low costs, solvent-free operation, and procedural simplicity [21]. When coupled with Gas Chromatography–Mass Spectrometry (GC-MS), HS-SPME has become a powerful and routine tool for the identification and characterization of aroma profiles. This technique’s efficacy is demonstrated in diverse studies [22,23]. It is critical to emphasize the fundamental distinction between HS-SPME and exhaustive extraction techniques such as hydrodistillation. HS-SPME is a non-exhaustive, equilibrium-based sampling method designed to capture the volatile compounds readily released into the headspace under ambient or mild heating conditions, thereby providing a highly accurate representation of the aroma profile perceived by the human olfactory system [24]. This makes it the ideal choice for sensory-driven studies aiming to characterize the authentic, fresh aroma of biological samples without inducing thermal degradation or artifactual formation of compounds. In contrast, hydrodistillation is an exhaustive preparative technique designed to isolate the complete volatile fraction, including high-boiling-point compounds, through prolonged heating and co-distillation with water, resulting in an essential oil (EO) [25]. While hydrodistillation is indispensable for obtaining substantial quantities of material for downstream commercial applications (e.g., flavorant production, bioactivity testing, or product standardization), the aggressive thermal process can alter the native volatile profile through degradation, isomerization, or hydrolysis of heat-labile constituents [26]. Consequently, the VOC profiles generated by these two methods are inherently divergent and serve different purposes; HS-SPME reflects the sensorially relevant aroma, while hydrodistillation provides a complete extract for material production. Furthermore, HS-SPME transcends mere compound identification by enabling the dynamic monitoring of volatile compound behaviour under various conditions, including storage, processing, or environmental stress [27,28]. For instance, a recent study by López et al. effectively employed GC-MS to track the oxidative stability and molecular modifications of volatile compounds from oregano and hop essential oils in sunflower oil subjected to prolonged high-temperature heating (150 °C for 8 h) [29]. Their work demonstrated how key terpenes, such as terpinen-4-ol and β-myrcene, were monitored throughout the thermal process, revealing their persistence and contribution to antioxidant protection. This aligns perfectly with the capability of HS-SPME to monitor temporal changes, degradation kinetics, and transformation pathways of aroma-active molecules, making it a powerful tool for investigating postharvest physiology, processing impacts, and shelf-life stability.

While HS-SPME-GC-MS enables precise characterization of volatile profiles, determining the sensory significance of identified compounds requires evaluating their perceptual impact. The Odor Activity Value (OAV), defined as the ratio of a compound’s concentration to its ortho nasal detection threshold [30], provides a quantitative metric to prioritize aroma-active compounds. The strategic selection and breeding of cultivars represent a critical endeavour in agricultural science, enabling the systematic enhancement of commercially and nutritionally significant traits. This approach is paramount for developing tailored varieties that meet evolving consumer preferences, environmental challenges, and market opportunities.

However, a comprehensive and sensory-driven characterization of the aroma-active compounds responsible for the distinct flavour profiles across different KC cultivars remains unexplored. Specifically, the key odorants defining cultivar uniqueness and their potential interactions with olfactory receptors have not been elucidated, creating a knowledge gap that significantly hinders targeted quality improvement through breeding programs. To address this, the present study was designed with three interconnected objectives: first, to characterize and compare the key aroma-active compounds in three distinct cultivars of KC fruits using an integrated approach combining HS-SPME/GC–MS with ROAV analysis; second, to elucidate the interaction mechanisms between identified key aroma compounds and human olfactory receptors through molecular docking simulations, thereby providing a molecular-level understanding of perceived aroma attributes; and third, to correlate instrumental aroma analysis with sensory evaluation results, establishing holistic aroma profiles for each cultivar and identifying potential biomarkers for sensory-driven quality breeding. We hypothesize that significant differences exist in the type and concentration of key aroma-active compounds among cultivars, and that these differences can be explained at the molecular level through specific ligand-receptor interactions. Ultimately, this research aims to establish a scientific foundation for the quality assessment and genetic improvement of KC as an emerging fruit crop with significant commercial potential.

## 2. Materials and Methods

### 2.1. Plant Materials

The plant materials utilized in this study were selected from germplasm resources collected and maintained by our research group. Mature fruits of three KC accessions (F023, F054, F055) were harvested from their respective production regions during the October-November period of the 2023 growing season. To ensure representativeness, fruits were collected from at least 8 healthy adult plants per accession. The average fruit weight ranged from 0.6 to 0.8 kg across accessions, with F023, F054, and F055 exhibiting comparable sizes within this range. Morphologically, the fruits are aggregate structures with an irregular spherical shape and a rough skin texture. The diameter varies from 10 to 15 cm. The skin coloration is accession-specific: F023 exhibits bright red, F054 displays purplish red, and F055 features dark red hues. The flesh is white and juicy, containing multiple embedded seeds. All plant materials were taxonomically authenticated as *Kadsura coccinea* (Lem.) A.C. Smith, a species within the genus Kadsuraof the family Schisandraceae, by Associate Research Fellow Xujun Wang of the Hunan Academy of Forestry Sciences.

**Figure 1:**
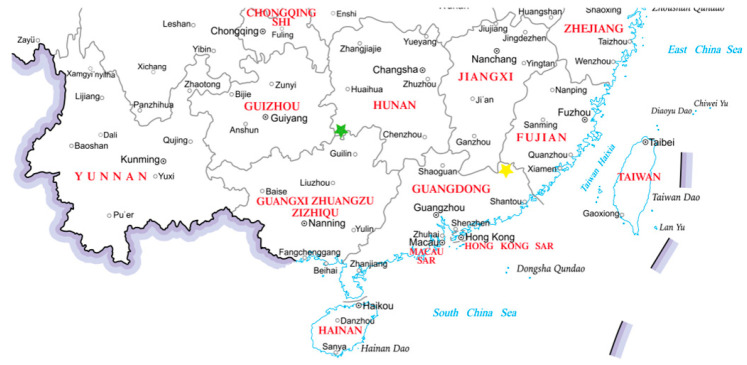
Regional map of sample collection sites. Yellow star denotes Pingyuan County, while green star represents Tongdao County.

The two production regions are situated in distinct agro-climatic zones, which may contribute to phenotypic and phytochemical variations among the accessions. Tongdao County, Hunan (F023, F054) is characterized by a mid-subtropical monsoon climate with an average annual temperature of 16.3 °C and abundant rainfall (≈1300 mm). Its acidic red soil (pH 4.5–5.5) is typical of the hilly terrain of southern China. In contrast, Pingyuan County, Guangdong (F055) experiences a southern subtropical climate with higher average annual temperatures (20.8 °C) and significantly greater precipitation (≈1600 mm). The region features lateritic red soils with slightly higher pH (5.0–6.0) and distinct mineral profiles. Geographical distribution of sampling locations was shown in Figure 1. While a comprehensive soil and climate analysis across all cultivation sites was beyond the scope of this study, these known environmental differences provide an important context for interpreting the observed chemotypic variations in volatile profiles among the accessions. Consequently, the findings of this study reflect the combined influence of genetic background and potential environmental effects, offering a realistic representation of the fruit’s aroma composition under diverse growing conditions.

Based on systematic sensory evaluation conducted by our research team, the three accessions exhibited significant divergence in aroma intensity: F023 was characterized by a relatively weak aroma, F054 presented a moderate aromatic profile, while F055 demonstrated the most intense and complex fragrance. Notably, F054 is the predominant cultivar in current commercial production and constitutes the major genotype circulating in the marketplace.

### 2.2. Sample Preparation

Fresh KC tissues were harvested from remote production sites and transported to the laboratory under refrigerated conditions (4 °C with ice packs) to minimize metabolic activity during transit. The transport duration was approximately 7 h due to geographical distances. Upon arrival, samples were immediately subjected to cryogenic grinding with a liquid nitrogen-cooled cryogenic planetary ball mill (QM-2L, JieChenLab, Shaoxing, China). This approach preserves thermo-labile aroma constituents by instantaneously halting enzymatic activity and suppressing molecular mobility. Powders were freeze-dried at −50 °C/0.1 mbar for 72 h to preserve thermo-labile volatiles, referencing stability benchmarks for lychee matrices [31]. Dried samples were immediately transferred to gas-impermeable laminated bags with oxygen scavengers (Ageless^®^ ZP series, Mitsubishi Gas Chemical, Japan), flushed with nitrogen, and sealed under negative pressure. Each bag was assigned a unique alphanumeric code to ensure traceability. A homogenized QC blend was prepared by combining equal masses of powdered materials from fruits of three accessions (F023, F054, F055). The mixture underwent vortex blending (3000 rpm, 10 min) followed by sonication-assisted homogenization (40 kHz, 15 min) to achieve particle size uniformity (D90 < 50 μm). For headspace analysis, 0.5 g (±0.001 g) of each sample and the QC blend were precisely weighed into silanized glass vials.

### 2.3. HS-SPME-GC-MS Analysis

The SPME fibre assembly (50/30 μm DVB/CAR/PDMS, Anpel, Shanghai, China) was conditioned by inserting it into the GC injector port (240 °C) for 40 min prior to initial use to remove contaminants. For extraction, 0.5 g of cryogenically ground sample was placed in a headspace vial. The vial was incubated at 50 °C for 40 min to achieve matrix-volatile equilibrium. Chromatographic separation was performed using GC-MS spectrometer (QP2010, Shimadzu, Tokyo, Japan) equipped with an HP-88 capillary column (100 m × 0.25 mm, 0.20 μm; Agilent, Santa Clara, CA, USA) with helium (99.999% purity) as carrier gas at a constant flow of 1.37 mL/min. The injector temperature was maintained at 240 °C in split mode (split ratio 5:1). The oven temperature program was as follows: 60 °C (hold 5 min), ramp to 140 °C at 3 °C/min (hold 5 min), then to 210 °C at 5 °C/min (hold 10 min), and finally to 240 °C at 5 °C/min (hold 10 min). The transfer line temperature was 220 °C. Mass spectrometric detection employed electron ionization (70 eV) at 200 °C source temperature. Full-scan data acquisition (*m*/*z* 50–600) at 0.50 scans/s was performed with solvent delay of 9.5 min. Compound identification was achieved by comparing mass spectra with NIST 14, 14s, 17, and 17s libraries (match factor > 800). All experimental procedures were performed in triplicate to ensure statistical robustness and reproducibility. The relative abundance of each volatile compound was quantified using the peak area normalization method, whereby the integrated chromatographic peak area of individual compounds was expressed as a percentage of the total peak area across all detected volatiles.

### 2.4. Relative Odour Activity Value Assessment

Odor threshold values (OTs) were obtained from the VCF Online 16.10 database (https://www.vcf-online.nl/VcfHome.cfm, accessed on 15 September 2025). Thresholds in air (OA) were prioritized for use in this study. When unavailable, odour thresholds in water (OW) were applied. For compounds with multiple reported values, the arithmetic mean was used as the final representative threshold. The contribution of individual volatile compounds to the overall aroma profile was quantified using the Relative Odor Activity Value (ROAV) approach [32]. This method evaluates the perceptual significance of volatiles by integrating concentration data with olfactory thresholds, defined as

$$ROAVi=(\frac{C\%i/Ti}{C\%max/Tmax})\times 100$$

where *C*_%_*_i_* = the relative concentration of compound *i* (peak area % via normalization); *T_i_* = the odor threshold of compound i (mg/kg in water); *C_%max_* = the highest relative concentration among all volatiles; and *T_max_* = the odor threshold of the dominant compound. ROAV ≥ 1.0 indicates key aroma compounds (decisive impact on overall flavour); 0.1 ≤ ROAV < 1.0 indicates modifier compounds (nuance-enhancing effects).

### 2.5. Molecular Docking

Based on the target odorant molecules, ten potential human olfactory receptors (ORs) were selected, namely OR1A1 [33], OR1G1 [34], OR2W1 [35], OR5AN1 [36], OR2AG1 [37], OR10G4 [38], OR51E2 (PDB: 8F76) [39], OR5M3 [40], OR7D4 [41], OR8D1 [34], OR5P3 [42]. Due to the lack of experimentally determined structures for target ORs (except OR51E2), high-confidence AlphaFold2 prediction models were utilized for molecular docking studies [43]. Structural templates for the target proteins were acquired from the UniProt knowledgebase (https://www.uniprot.org/, accessed on 5 September 2025) and the RCSB Protein Data Bank (https://www.rcsb.org/, accessed on 5 September 2025). All ligand structures, in the form of SDF format, were sourced from the PubChem chemical compound database (https://pubchem.ncbi.nlm.nih.gov/, accessed on 5 September 2025). The molecular docking studies were performed using the Schrödinger computational chemistry software suite (release 2021-2). The protein structures were prepared with the Protein Preparation Wizard module. This process involved the assignment of bond orders, addition of hydrogens, creation of disulfide bonds, and optimization of hydrogen bonding networks. The protonation states of ionizable residues were predicted at physiological pH (7.0 ± 2.0) using the integrated PROPKA tool. Ligand structures were prepared using the LigPrep module, which generated possible tautomers, protonation states, and stereoisomers at pH 7.0 ± 2.0. Energy minimization was performed with the OPLS4 force field to ensure proper geometry. The receptor grid generation was conducted using the Receptor Grid Generation tool. The centroid of the co-crystallized ligand (when available) or the predicted binding site residues was used to define the grid box (size: 36 × 36 × 36 Å). Default van der Waals scaling (1.0) and partial charge cutoff (0.25) were applied. Molecular docking was executed using the Glide module with Standard Precision (SP) mode for screening. The docking poses were evaluated based on the docking score. The top-ranked poses were selected for subsequent analysis and visualization using Maestro 12.8 and Protein Ligand Interaction Profiler (PLIP) (https://plip-tool.biotec.tu-dresden.de/plip-web/plip/index, accessed on 10 September 2025) [44].

### 2.6. Descriptive Sensory Analysis

Written informed consent was obtained from all participants prior to the commencement of the study. The consent form explicitly outlined the study procedures and guaranteed the right to withdraw at any time without penalty. All participant data were anonymized and handled confidentially.

A panel of twelve assessors (6 males, 6 females; aged 22–35 years) was recruited and rigorously screened in accordance with ISO 8586:2012 standards. Screening included basic taste identification and odour detection threshold tests using a series of aqueous solutions and odorants relevant to the KC fruit profile (e.g., citric acid for sourness, linalool for floral notes). Following successful screening, the panel underwent 40 h of standardized training over four weeks to develop a consensus lexicon and scale the intensity of attributes. Seven key sensory attributes were selected to describe the odour intensity of KC fruits (Table 1). Training sessions involved repeated exposure to these reference standards to calibrate the panel’s understanding of each attribute and the full 0–10 intensity scale (0 = absent; 5 = moderate; 10 = extremely strong).

Individual KC fruits of uniform size were peeled and homogenized. A 10 g aliquot of fruit pulp was placed into 150 mL odourless disposable plastic cups and sealed with a lid. Each cup was labelled with a unique random three-digit code. The coded samples were presented to the assessors in a dedicated sensory laboratory conforming to ISO 8589:2007 standards. The environment was strictly maintained at 25 ± 0.5 °C and 50 ± 5% relative humidity, with positive air pressure and ambient lighting to standardize testing conditions and prevent olfactory fatigue. The QDA protocol required assessors to evaluate each sample and score the perceived intensity of each of the seven attributes on the 0–10 scale. Each assessor evaluated all three cultivars (F023, F054, F055) in a randomized, balanced order across three independent sessions (replications), with a 24-h interval between sessions to prevent carry-over effects. Between samples, evaluators were asked to cleanse their palate with salt-free biscuits and deionized water (20 °C). Paper forms were used to collect data.

### 2.7. Statistical Analysis

Experimental data represent the mean ± SD of triplicate determinations (*n* = 3). Significant differences (*p* < 0.05) among groups were evaluated by one-way ANOVA followed by Duncan’s or Turkey’s post hoc test using SPSS 26.0.

## 3. Results and Discussion

### 3.1. VOCs Analysis in Fruits

**Figure 2:**
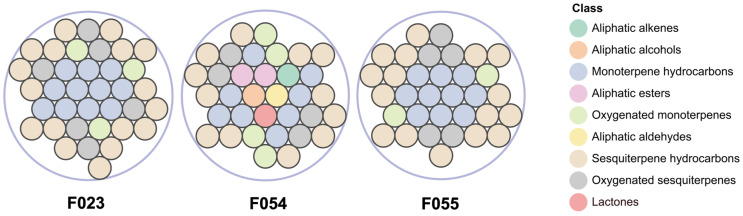
Comparative analysis of VOC categories among three KC accessions. Total compounds identified: F023 (37), F054 (34), F055 (33).

HS-SPME-GC-MS analysis identified 49 VOCs across three KC accessions (F023, F054, F055), categorized into 9 chemical classes based on structural characteristics (Figure 2): 12 monoterpene hydrocarbons, 20 sesquiterpene hydrocarbons, 11 oxygenated monoterpenes, 2 aliphatic esters, 1 aliphatic alkene, 1 aliphatic alcohol, 1 aliphatic aldehyde, and 1 lactone. A total of 19 volatile compounds were shared among the three accessions. Accession F054 contained 7 unique constituents, including 6 non-terpenoid compounds. Accessions F055 and F023 exhibited 4 and 1 accession-specific terpenoids, respectively (Table 2). The current investigation identified 49 volatile compounds, with 20 demonstrating substantial overlap with prior reports [14], including characteristic terpenoids such as α-pinene, β-pinene, α-terpinene, and β-caryophyllene. Divergently, two high-abundance compounds previously detected in fruit pulp—γ-curcumene (12.55%) and α-bisabolol (6.17%)—were not observed. This discrepancy arises from: (i) the whole-fruit homogenization approach diluting pulp-specific volatiles, thereby reducing headspace concentrations below detection thresholds; and (ii) the limited volatility of high-boiling-point compounds (e.g., γ-curcumene; b.p. 265–268 °C) under HS-SPME conditions, contrasting with hydro-distillation’s efficacy in recovering such thermostable terpenes. Although cross-study comparisons remain constrained by methodological disparities (e.g., hydro-distillation vs. HS-SPME) and potential genotypic differences, the consistent detection of fruit-like volatiles—specifically aliphatic esters and lactones—exclusively in F054 across biological replicates confirms its unique chemotype. This accession-specific signature suggests a genetically encoded metabolic predisposition potentially amplified by post-harvest enzymatic activity, independent of extraction artifacts [45].

Our results position F054 as a sensorially distinct cultivar characterized by six signature volatiles. The sensory impact of these compounds follows a synergistic model: butyl butanoate and butyl caproate establish the primary fruity frame (pineapple and apple) [46,47], while γ-dodecalactone introduces lactone-derived sweetness (milky, creamy and coconut) that exceeds the additive effects of individual components [48]. The C9 aldehyde (nonanal) operates sub-threshold as a flavour modifier, amplifying perceived complexity through waxy-citrus interactions [49]. Meanwhile, the C6 alcohol contribute green-herbaceous accents that complete the aroma profile [50,51]. Although 1,3,5-heptatriene has not been definitively linked to specific flavour attributes in any study, its methylated derivatives are recurrently identified in volatile profiles of herbal species and are postulated to impart herbaceous and floral nuances to complex aroma matrices [52,53]. The dominance of γ-dodecalactone in F054 presents a striking parallel to its role in strawberry [54], yet its occurrence in Schisandraceae fruits is unprecedented. Prior studies on related *Schisandra chinensis* identified α-pinene and limonene as key volatiles [18], but lactones were conspicuously absent—highlighting a unique biosynthetic pathway activation in KC. This divergence may arise from differential expression of enzymes related to lactone synthesis, suggesting KC-specific genetic regulation. Experimental evidence confirms that 9-lipoxygenase and epoxide hydrolase 2 genes directly regulate the biosynthetic flux of mango lactones through the oxylipin pathway, significantly altering their endogenous accumulation levels [55]. Transcriptional profiling confirms that FaFAD1modulates the flux of hydroxyl fatty acid precursors in the lactone biosynthesis pathway, directly governing γ-decalactone (C10) and γ-dodecalactone (C12) accumulation in strawberry [56,57,58].

Quantitative results show that sesquiterpene hydrocarbons constituted the most abundant chemical class across all accessions, ranging from 65.2% to 78.4% of total VOC abundance. F023 showed the highest sesquiterpene accumulation (78.4%), primarily driven by exceptionally high β-caryophyllene (34.02%) and humulene (0.19%) levels. These two compounds exhibited a progressively decreasing trend across the accessions (F023→F054→F055), demonstrating a statistically significant negative correlation with the aromatic evolution pattern of KC fruits (*p* < 0.001). F054 exhibited dual terpenoid/non-terpenoid profile with elevated α-pinene (9.24%) and non-terpenoid compounds (totalling 1.03%). F055 showed distinct sesquiterpene pattern with high γ-maaliene (12.50%) and β-elemene (9.73%), and they showed an increasing trend and were positively correlated with odour intensity (*p* < 0.001). In F055, six quantitatively dominant VOCs (β-selinene: 10.87%, γ-maaliene: 12.50%, β-pinene: 10.32%, γ-muurolene: 10.37%, β-elemene: 9.73%, and β-Caryophyllene: 12.80%) collectively constitute 66.59% of total volatiles. Their synergistic interactions, particularly between sesquiterpenes, likely underlie the complex, intense fragrance (Section 2.1). The antagonism between β-caryophyllene (F023-dominant) and β-pinene (F055-dominant) suggests competitive inhibition in terpene synthase pathways. While such metabolite trade-offs are documented in grape, tomato and freesia [59,60,61], our report provides the first evidence in Schisandraceae. β-Caryophyllene and β-pinene engage in metabolic antagonism through competition for the shared IPP/DMAPP precursor pool and membrane-bound catalytic sites. Their flux partitioning is co-determined by the FDPS/GPPS enzymatic activity ratio and cultivar-specific environmental adaptations [62,63]. This biochemical divergence likely reflects adaptive responses to native environments. F055’s Guangdong provenance (high humidity and temperature) may favour the expression and release of monoterpene oxidation products [64], whereas F023’s Hunan highland origin selects for sesquiterpene-based cold resistance [65]. A critical question arising from these findings is whether the distinct chemotypic profiles of the three cultivars are driven primarily by genetic differences or by environmental influences (e.g., climate, soil) of their respective growing regions. While the potential contribution of environmental factors cannot be entirely ruled out, the evidence suggests that genetic background is the predominant driver. The most compelling support for this conclusion comes from the comparison between F023 and F054, which were grown in the same geographical location (Tongdao County, Hunan) and were therefore subjected to identical climatic and edaphic conditions. Despite this environmental uniformity, their volatile compositions were fundamentally different: F023 exhibited a sesquiterpene-dominant (e.g., β-caryophyllene) and woody–pungent profile, whereas F054 was characterized by a fruity–floral signature rich in esters and lactones (e.g., γ-dodecalactone). This stark contrast under a shared environment strongly indicates that the divergence is under robust genetic control, likely involving differential expression or activity of key biosynthetic enzymes.

The role of environment may be more modulatory, potentially explaining certain quantitative variations, such as the elevated levels of monoterpene oxidation products in F055, which was grown in the warmer and more humid climate of Guangdong Province. To definitively apportion the variance between genetic and environmental effects, future studies should employ a common garden experimental design, cultivating all genotypes in a controlled environment or across multiple locations.

### 3.2. ROAV-Driven Comparative Analysis of Odour Activity Characteristics Among Varieties

ROAV analysis was employed to decipher the contribution of individual volatile compounds to the overall aroma profile of each KC accession. Compounds with ROAV ≥ 1 were defined as key aroma contributors, while those with ROAV = 0 were sensorially negligible (either absent or below perceptual thresholds). The contribution of VOCs in KC fruit to overall odour is shown in Table 3. F023 exhibited a warm, resinous-woody character dominated by humulene (ROAV = 100.0; odour threshold: 0.16 mg/m^3^), which imparts balsamic and earthy notes, alongside β-myrcene (ROAV = 97.6; threshold: 0.061 mg/m^3^) contributing herbal-fruity nuances. This dominance suggests high terpenoid synthase activity in F023 [66]. The high ROAV of D-limonene (31.1) further added a subtle citrus undertone, absent in other accessions. F054 demonstrated a fruity–floral signature, primarily driven by β-myrcene (ROAV = 100.0) and γ-dodecalactone (ROAV = 73.0; threshold: 0.0048 mg/m^3^), whose ultra-low threshold explains its outsized sensory impact despite moderate concentration (0.24%). Butyl butanoate (ROAV = 18.8) and linalool (ROAV = 23.6) amplified the fruity and floral layers, synergistically reinforcing the “fruity-sweet” character. Notably, γ-dodecalactone—undetected in F023/F055—may serve as a chemotaxonomic marker for F054. F055 was characterized by a fresh herbaceous-pine profile, with β-pinene (ROAV = 100.0; threshold: 0.14 mg/m^3^) as the dominant contributor, delivering pine and resinous notes. β-Myrcene (ROAV = 88.5) provided supporting green-herbal accents, while β-phellandrene (ROAV = 4.2; detected only in F055) added minty nuances. This correlates with the “fresh-green” sensory evaluation. Cross-variety patterns revealed β-Myrcene was a universal high-impact compound across all accessions (ROAV > 88), attributed to its exceptionally low threshold (0.061 mg/m^3^), enabling dominance even at sub-percentage concentrations; Humulene showed accession-dependent effects, via dominant in F023 (ROAV = 100) but moderate in F054/F055 (ROAV = 51.9/31.0), potentially linked to varietal terpene synthase isoforms; Synergistic interactions were evident in F055, where β-pinene and β-myrcene collectively accounted for 188.5% of the top ROAV, suggesting additive potentiation of herbaceous notes. ROAV analysis resolved discrepancies between compound abundance and sensory impact. For instance, β-Caryophyllene showed high concentration in F023 (34.02%) but moderate ROAV (35.6), indicating limited perceptual influence despite quantitative dominance; Nonanal (ROAV = 0.8 in F054) and 1-hexanol (ROAV = 0.3) acted as background modifiers but were non-decisive individually. Their collective role in aroma rounding warrants further study. Accession F054 is identified as a premium candidate for targeted breeding and commercialization, owing to its genetically encoded synthesis of fruity-scented volatiles (e.g., γ-dodecalactone, butyl esters) and well-balanced terpenoid backbone.

### 3.3. Molecular Docking Elucidates Binding Patterns of Key Odorants

**Figure 3:**
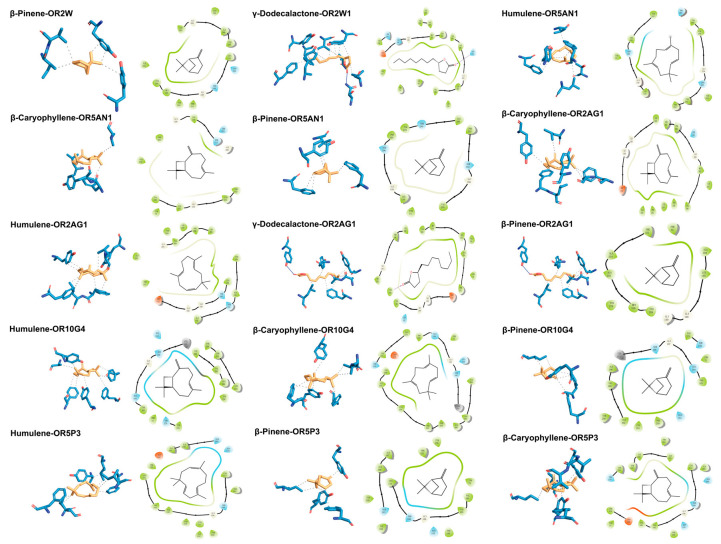
Two-dimensional and 3D binding poses of olfactory receptors with key odorant molecules; 3D poses generated by PLIP: hydrophobic interactions (black dashed lines), hydrogen bonds (dark blue solid lines); 2D poses generated by Maestro: hydrogen bonds (rose red arrows).

To mechanistically decipher the odour contribution of ROAV-identified key compounds, molecular docking simulations were performed between eight target odorants—five shared across cultivars (β-Pinene, β-Myrcene, β-Ocimene, β-Caryophyllene, and Humulene) and three unique to F054 (Butyl butanoate, Linalool, γ-Dodecalactone)—and 11 human ORs. The docking protocol followed Section 2.5, with binding energies < −5 kcal/mol indicating stable complexes (Wu et al., 2025) [67]. Shared terpenoids exhibited superior binding affinity. β-Pinene, Humulene, and β-Caryophyllene showed the lowest binding energies (range: −7.439 to −5.875 kcal/mol), forming stable complexes with multiple ORs (e.g., OR2AG1, OR5AN1, OR10G4) (Table 4). This multi-receptor engagement suggests their role as universal chemical cues for KC’s core flavour profile. OR5AN1 is currently the only olfactory receptor exclusively reported to recognize musky odorants, with Tyr102−Tyr279 identified as its key recognition residues. Humulene, β-caryophyllene, and β-pinene—structurally analogous to musky compounds due to their macrocyclic/polycyclic scaffolds—demonstrate binding to OR5AN1’s ligand pocket, engaging critical residues including Phe105 and Tyr279 in molecular docking simulations [36]. γ-Dodecalactone uniquely formed hydrogen bonds. As the sole F054-specific compound with significant ROAV (73.0), it established H-bonds with ASN155 of OR2W1 (binding energy: −6.422 kcal/mol), potentially underpinning its cultivar-distinct sensory impact. OR2W1 is recognized as the olfactory receptor with the broadest ligand recognition spectrum reported to date. Our molecular docking results demonstrate that γ-dodecalactone forms a high-affinity hydrogen bond with ASN155 of OR2W1, providing mechanistic validation beyond empirical correlation. Complementary in vitro functional assays confirm OR2W1’s specificity for lactone compounds, with cell-based agonist screening identifying γ-lactones as potent activators (75% hit rate) [35]. Hydrophobic interactions dominated binding mechanisms. Across all 15 stable complexes, hydrophobic packing accounted for 86.7% of interactions (13 complexes), involving residues such as TYR104, PHE251, and VAL207. This aligns with the hydrophobic nature of both odorants and OR binding pockets [36,68,69]. Visualization of high-affinity complexes confirmed these patterns (Figure 3). For instance, β-Pinene occupied a hydrophobic sub-pocket of OR2W1 via hydrophobic Interaction contacts with TYR104, ILE206, VAL207 and TYR259; γ-Dodecalactone adopted a pose where its lactone ring formed H-bonds with ASN155 while its alkyl chain engaged in hydrophobic stacking with PHE73, TYR104, LEU159, VAL207, PHE251, ILE255, TYR259 and TYR278. These structural insights provide a molecular basis for the ROAV-driven odorant prioritization, highlighting how shared terpenoids stabilize KC’s foundational aroma, while γ-dodecalactone drives F054’s uniqueness through dual interaction modes.

### 3.4. Multivariate Analysis of Sensory Profiles and Volatile Compounds in KC Cultivars

**Figure 4:**
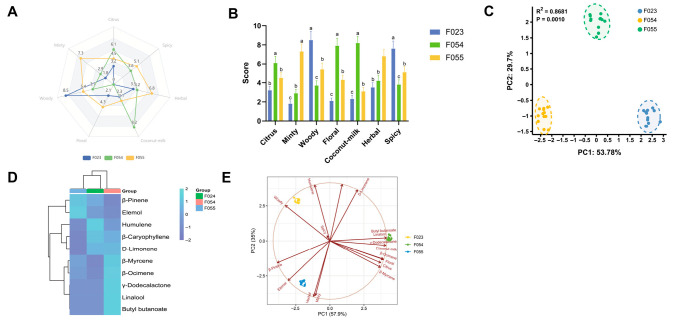
Sensory attribute analysis of three kc varieties. (**A**) Radar map of sensory attribute descriptors; (**B**) Difference analysis of sensory attributes; (**C**) PCA of sensory attributes of different KC varieties; (**D**) Cluster Heatmap of Key Aroma Compounds in Three KC Cultivars; (**E**) PCA loading plot for sensory attributes combined with key odour molecules.

The flavour characteristics of three KC cultivars (F023, F054, F055) were systematically evaluated through descriptive sensory analysis and principal component analysis (PCA), revealing distinct flavour profiles and their underlying chemical drivers. Sensory attribute intensity patterns visualized through radar plot (Figure 4A) demonstrated cultivar-specific dominance: F054 exhibited pronounced coconut-milk and floral attributes (scores 8.2 ± 0.7 and 7.9 ± 0.8, respectively), establishing its “sweet-aromatic” signature. F055 showed dominance in citrus and minty dimensions (7.3 ± 0.8 and 6.8 ± 0.7), characterizing its “fresh-fruity” profile. F023 displayed elevated woody and spicy intensities (8.5 ± 0.9 and 7.6 ± 0.8) with minimal fruity notes, defining its “woody-pungent” character. Statistical validation through ANOVA with Tukey’s post hoc test (Figure 4B) confirmed significant inter-cultivar differences (*p* < 0.001) across all attributes. Coconut-milk intensity followed F054 > F055 ≈ F023 (a, b, c grouping), while Woody intensity showed F023 > F055 > F054 (a, b, c grouping). This clear segregation indicates genetic regulation of flavour compound biosynthesis pathways.

PCA of sensory attributes (Figure 4C) explained 83.48% of the total variance (PC1: 53.78%, PC2: 29.7%) with high statistical significance (*p* = 0.0010). The score plot revealed F054 clustering along the PC1-positive axis versus F023 along the negative axis, indicating orthogonal flavour profiles. F055 separated along PC2, suggesting that its unique flavour architecture is distinct from the PC1-driven “sweet-woody” continuum. The cluster heatmap analysis, based on Z-score normalized values, provided a comprehensive visualization of the relative abundance patterns of key aroma-active compounds across three distinct KC cultivars (Figure 4D). The analysis revealed pronounced differences in the expression profiles of terpenoid compounds, which emerged as the primary discriminators among cultivars. β-Pinene exhibited markedly elevated expression in cultivar F055, characterizing it as a signature compound for this genotype, whereas it showed suppressed levels in F023 and F054. Conversely, eucalyptol demonstrated highest abundance in F023, suggesting its role as a potential biomarker for this cultivar, with minimal expression in F055. Humulene displayed relatively uniform expression across all cultivars but with a slight elevation in F054, indicating moderate contribution to its aroma profile. In contrast, ester compounds, such as γ-dodecalactone and butyl butanoate, displayed more conserved expression patterns; γ-dodecalactone was consistently low across all cultivars, forming a distinct cluster separate from terpenoids, which implies divergent regulatory mechanisms or biosynthetic pathways. Butyl butanoate showed a subtle increase in F054 but remained near mean levels in others. Additional compounds, including aldehydes and alcohols like hexanal and phenethyl alcohol, exhibited minimal variation, indicating their secondary role in cultivar differentiation. Hierarchical clustering of compounds (row-wise) yielded two primary clusters: one encompassing most terpenoids (e.g., β-pinene, eucalyptol, humulene) with similar expression profiles, suggesting co-regulation under shared biosynthetic pathways, and another comprising esters (e.g., γ-dodecalactone) with distinct behaviour, reinforcing the metabolic divergence between these compound classes. Cultivar clustering (column-wise) showed that F054 and F055 grouped together first, subsequently clustering with F024, indicating that F054 and F055 share more similar aroma compound compositions, while F024 possesses a unique chemotype. This clustering pattern aligns with prior PCA results, corroborating the phenotypic divergence observed in sensory and chemical analyses. Joint PCA loading plot (Figure 4E) integrating sensory and chemical data revealed compound-attribute correlations driving cultivar differentiation. γ-Dodecalactone showed vector alignment with coconut-milk attribute (θ < 10°), confirming its role as key odorant for F054’s signature flavour, consistent with prior ROAV analysis (ROAV = 73.0) and molecular docking results (−6.422 kcal/mol binding energy). Similarly, β-Caryophyllene spatially correlated with woody attribute (r > 0.9), explaining F023’s woody character. The combined model explained 83.5% variance (PC1: 57.9% + PC2: 25.6%), demonstrating enhanced explanatory power through chemical data integration. The covariance patterns in loading plots suggest modular organization of flavour traits: “coconut-floral” (F054), “citrus-minty” (F055), and “woody-spicy” (F023) modules likely reflect coordinated regulation of biosynthetic pathways. PC1 appears governed by genetic background regulating terpene synthase expression, while PC2 may involve environment-responsive pathways (e.g., monoterpene oxidation in F055’s growing region). Practical implications include using PC1 > 0.5 with γ-dodecalactone dominance as selection criterion for sweet-aromatic cultivars. Future research should employ GC-O-MS to identify odour-active compounds corresponding to each sensory vector and conduct consumer acceptance tests to validate preference patterns. In conclusion, multivariate analysis revealed three distinct flavour phenotypes in KC cultivars governed by coordinated expression of flavour compound modules. The strong sensory–chemical correlations provide a framework for targeted flavour breeding and product development.

It is important to contextualize the analytical approach taken in this study. The cryogenic grinding and subsequent storage under a nitrogen atmosphere in gas-impermeable packaging were employed to effectively ‘freeze’ the metabolic state of the fruit at the moment of sampling. This stringent preservation method was essential to establish a definitive baseline volatile profile for each cultivar, minimizing post-harvest biochemical changes and oxidative degradation that would otherwise confound the assessment of genotypic differences [70]. Consequently, the aroma profiles reported herein represent the innate potential of each cultivar under ideal preservation conditions.

However, as astutely noted, this controlled environment does not reflect the practical realities of a commercial supply chain, where fruits are typically stored in permeable packaging at ambient or refrigerated temperatures. Under such conditions, the key aroma-active compounds identified—particularly the highly volatile esters (e.g., ethyl butanoate) and oxidation-sensitive terpenes (e.g., β-pinene)—are susceptible to rapid degradation through enzymatic activity, oxidation, and evaporation [71]. Therefore, the sensory experience of the consumer, which is the ultimate determinant of commercial success, will be governed by the stability of this aroma portfolio during postharvest handling. This presents a critical avenue for future research. Our study provides the essential reference point for such investigations. Subsequent work should focus on monitoring the depletion of these key odorants (tracked via OAV) under realistic storage scenarios. Evaluating the efficacy of practical preservation technologies, such as modified atmosphere packaging, edible coatings, or cold chain logistics, in maintaining the desirable aroma attributes of cultivars like F054 will be vital. Ultimately, integrating genotypic selection with optimized postharvest protocols will be key to delivering the unique sensory promise of KC to the consumer.

## 4. Conclusions

This study establishes that the distinct aroma profiles of KC cultivars arise from genetically encoded volatile signatures, which were validated through integrated chemosensory analysis. F054 exhibits a premium fruity–floral character driven by γ-dodecalactone, which forms high-affinity hydrogen bonds with ASN155 of the olfactory receptor OR2W1, as revealed by molecular docking. F023’s woody–pungent profile correlates with sesquiterpene dominance (humulene), while F055’s herbaceous freshness stems from β-pinene and monoterpene oxides. PCA of sensory and chemical data cumulatively explained 83.5% of the variance. Sensory quantification confirmed F054’s superiority in coconut-milk (8.2 ± 0.7) and floral (7.9 ± 0.8) intensities, contrasting with F023’s peak woody (8.5 ± 0.9) and spicy (7.6 ± 0.8) attributes. Joint PCA loadings spatially co-localized γ-dodecalactone with coconut milk (θ < 10°) and β-caryophyllene with woody notes (r > 0.9), mechanistically linking chemistry to perception. These findings provide actionable targets for flavour breeding: γ-dodecalactone synthase upregulation enhances fruity complexity, while OR2W1-binding affinity screening (ΔG < −6.0 kcal/mol) enables precise selection of high-aroma genotypes, positioning KC as a novel flavour-focused fruit crop.

## Figures and Tables

**Table 1:** Sensory lexicon generated for descriptive sensory analysis of KC fruit odour.

Attributes	Reference	Definition
Citrus	0.01% D-Limonene in PG	Fresh, peel-like zest reminiscent of lemon or orange.
Minty	0.01% L-Menthol in water	Cooling, herbal note with a refreshing sensation.
Woody	0.1% Cedrol in PG	Resinous, bark-like aroma evoking pine or sandalwood.
Floral	0.02% Linalool in PG	Sweet, blossom-like scent similar to rose or jasmine.
Coconut-milk	Coconut juice	Creamy, lactonic sweetness akin to coconut or peach.
Herbal	0.05% 1,8-Cineole in PG	Green, medicinal character reminiscent of eucalyptus or grass.
Spicy	0.02% eugenol in PG	Pungent, warm note suggestive of pepper or clove.

Note: PG refers to Propylene Glycol, the solvent in which the odour thresholds were determined.

**Table 2:** Volatile organic compound composition in fruits of different KC accessions.

No.	Retention Time	Name	Molecular Formula	Percentage Composition (%)		
				F023	F054	F055
1	8.184	1,3,5-Heptatriene,(E,E)-	C_7_H_10_	ND	0.36 ± 0.14	ND
2	11.500	1-Hexanol	C_6_H_14_O	ND	0.16 ± 0.08	ND
3	14.207	α-Thujene	C_10_H_16_	0.11 ± 0.01 ^b^	ND	0.26 ± 0.04 ^a^
4	14.626	α-Pinene	C_10_H_16_	4.55 ± 0.65 ^b^	9.24 ± 1.27 ^a^	2.52 ± 0.50 ^b^
5	16.503	Sabinene	C_10_H_16_	0.55 ± 0.08 ^a^	0.13 ± 0.02 ^b^	ND
6	17.009	β-Pinene	C_10_H_16_	8.05 ± 1.21 ^b^	3.50 ± 0.57 ^c^	10.32 ± 4.32 ^a^
7	17.364	β-Myrcene	C_10_H_16_	3.79 ± 0.47 ^a^	4.17 ± 0.45 ^a^	3.98 ± 0.32 ^a^
8	17.539	Butyl butanoate	C_8_H_16_O_2_	ND	0.36 ± 0.10	ND
9	18.730	α-Terpinene	C_10_H_16_	0.08 ± 0.01 ^b^	ND	0.15 ± 0.01 ^a^
10	19.122	P-Cymene	C_10_H_14_	0.07 ± 0.01 ^a^	ND	0.06 ± 0.003 ^a^
11	19.367	D-Limonene	C_10_H_16_	0.89 ± 0.10 ^a^	0.93 ± 0.12 ^a^	ND
12	19.575	β-Phellandrene	C_10_H_16_	ND	ND	1.54 ± 0.11
13	20.151	β-Ocimene	C_10_H_16_	0.31 ± 0.02 ^b^	0.47 ± 0.02 ^a^	0.37 ± 0.05 ^b^
14	20.838	γ-Terpinene	C_10_H_16_	0.16 ± 0.03 ^b^	0.06 ± 0.02 ^c^	0.28 ± 0.01 ^a^
15	22.389	Terpinolene	C_10_H_16_	0.17 ± 0.02 ^b^	0.11 ± 0.01 ^c^	0.33 ± 0.02 ^a^
16	23.068	Linalool	C_10_H_18_O	ND	0.21 ± 0.04	ND
17	23.287	Nonanal	C_9_H_18_O	ND	0.13 ± 0.05	ND
18	26.263	Terpinen-4-ol	C_10_H_18_O	0.25 ± 0.06 ^a^	0.14 ± 0.03 ^b^	0.18 ± 0.02 ^ab^
19	26.440	Butyl caproate	C_10_H_20_O_2_	ND	0.30 ± 0.08	ND
20	26.723	α-Terpineol	C_10_H_18_O	0.22 ± 0.04 ^a^	0.12 ± 0.02 ^b^	ND
21	30.701	Bicyclogermacrene	C_15_H_24_	0.04 ± 0.02 ^b^	0.17 ± 0.02 ^a^	0.19 ± 0.01 ^a^
22	31.309	γ-Elemene	C_15_H_24_	0.35 ± 0.12 ^b^	2.00 ± 0.20 ^a^	1.72 ± 0.09 ^a^
23	31.780	α-Cubebene	C_15_H_24_	0.50 ± 0.09 ^b^	0.55 ± 0.03 ^b^	1.05 ± 0.03 ^a^
24	32.630	Ylangene	C_15_H_24_	ND	0.48 ± 0.02	ND
25	33.012	γ-Muurolene	C_15_H_24_	9.49 ± 0.42 ^a^	9.55 ± 1.10 ^a^	10.37 ± 0.26 ^a^
26	33.413	β-Elemen	C_15_H_24_	3.60 ± 0.88 ^b^	4.09 ± 0.45 ^b^	9.73 ± 0.46 ^a^
27	33.975	Isocaryophyllene	C_15_H_24_	0.79 ± 0.18	ND	ND
28	34.864	β-Caryophyllene	C_15_H_24_	34.02 ± 4.22 ^a^	28.54 ± 1.81 ^a^	12.80 ± 0.44 ^b^
29	35.067	Aciphyllene	C_15_H_24_	2.72 ± 0.38 ^a^	2.13 ± 0.24 ^a^	ND
30	35.319	α-Santoline alcohol	C_10_H_18_O	0.80 ± 0.09 ^a^	3.67 ± 2.02 ^a^	0.90 ± 0.05 ^a^
31	36.076	Humulene	C_15_H_24_	10.19 ± 1.24 ^a^	5.67 ± 0.71 ^b^	3.66 ± 0.11 ^b^
32	36.223	Alloaromadendrene	C_15_H_24_	1.05 ± 0.04 ^b^	ND	1.48 ± 0.10 ^a^
33	36.720	γ-Maaliene	C_15_H_24_	2.30 ± 1.60 ^c^	5.28 ± 0.63 ^b^	12.50 ± 0.88 ^a^
34	37.038	Germacrene D	C_15_H_24_	0.59 ± 0.07 ^b^	ND	2.06 ± 0.11 ^a^
35	37.592	α-Guaiene	C_15_H_24_	2.77 ± 0.70 ^b^	6.81 ± 0.92 ^a^	ND
36	37.728	β-Selinene	C_15_H_24_	ND	ND	10.87 ± 0.48
37	38.035	α-Selinene	C_15_H_24_	ND	ND	2.89 ± 0.19
38	38.277	γ-Cadinene	C_15_H_24_	1.31 ± 0 ^b^	ND	1.52 ± 0.07 ^a^
39	38.442	δ-Amorphene	C_15_H_24_	3.37 ± 0.44 ^b^	6.30 ± 0.92 ^a^	3.22 ± 0.46 ^b^
40	38.995	γ-Amorphene	C_15_H_24_	0.25 ± 0.06 ^b^	ND	0.42 ± 0.01 ^a^
41	39.150	α-Cadinene	C_15_H_24_	0.17 ± 0.02 ^b^	ND	0.36 ± 0.01 ^a^
42	39.594	Elemol	C_15_H_26_O	0.26 ± 0.12 ^b^	ND	0.74 ± 0.04 ^a^
43	39.900	E-Nerolidol	C_15_H_26_O	1.37 ± 0.32 ^a^	0.98 ± 0.20 ^a^	ND
44	41.228	Caryophyllene oxide	C_15_H_24_O	0.91 ± 0.17 ^a^	0.35 ± 0.03 ^b^	0.16 ± 0.01 ^b^
45	43.299	γ-Eudesmol	C_15_H_26_O	0.13 ± 0.03 ^a^	ND	0.18 ± 0.02 ^a^
46	43.769	t-Cadinol	C_15_H_26_O	0.29 ± 0.09 ^b^	0.71 ± 0.10 ^a^	0.33 ± 0.05 ^b^
47	44.348	t-Muurolol	C_15_H_26_O	1.11 ± 0.06 ^a^	0.41 ± 0.08 ^b^	ND
48	44.505	β-Selinenol	C_15_H_26_O	ND	ND	0.92 ± 0.10
49	45.087	γ-Dodecalactone	C_12_H_22_O_2_	ND	0.24 ± 0.07	ND

Note: Values are expressed as mean ± SD (*n* = 3). Means within a row with different superscript letters differ significantly according to Duncan’s multiple-range test (*p* < 0.05); ND = Not detected.

**Table 3:** Odour thresholds, ROAVs across varieties, and sensory profiles of volatile compounds.

Name	OA (mg/m^3^)	ROAV_F023	ROAV_F054	ROAV_F055	Odours
1-Hexanol	0.74	0	0.316	0	Almond, apple, banana, bread, cut grass
α-Pinene	7.9	0.904	1.711	0.433	Berry, citrus, floral, fruit, lemon
Sabinene	2	0.432	0.095	0	Citrus, green, herb, pepper, spice
β-Pinene	0.14 (OW)	90.307	36.577	100	Woody, terpentine
β-Myrcene	0.061	97.586	100	88.5	Balsamic, fruit, geranium, green, herb
Butyl butanoate	0.028	0	18.811	0	Floral
α-Terpinene	7.9	0.02	0	0.026	Berry, citrus, floral, fruit, lemon
P-Cymene	7.2	0.015	0	0.011	Citrus, fresh, fruit, gasoline, lemon
D-Limonene	0.045	31.066	30.239	0	Citrus, fresh, lemon, mint, orange
β-Phellandrene	0.5	0	0	4.178	Mint, spice, terpentine
β-Ocimene	0.034 (OW)	14.32	20.225	14.765	Herb, sweet
γ-Terpinene	37.5	0.007	0.002	0.01	Bitter, caramel, citrus, fruit, gasoline
Terpinolene	0.2	1.335	0.805	2.238	Citrus, floral, herb, pine, plastic
Linalool	0.013	0	23.636	0	Bergamot, coriander, floral, flower, grape
Nonanal	0.23	0	0.827	0	Aldehyde, beany, citrus, cucumber, fat
Terpinen-4-ol	0.59	0.665	0.347	0.414	Citrus, earth, floral, herb, must
Butyl caproate	0.7	0	0.627	0	Fruit, grass, green
α-Terpineol	0.41	0.843	0.428	0	Anise, citrus, floral, fresh, lily
β-Caryophyllene	1.5	35.618	27.838	11.578	Candy, citrus, clove, fried, green
Humulene	0.16 (OW)	100	51.854	31.034	Balsamic, carrot, hop, resin, soap
Elemol	0.1	4.084	0	10.038	Green, wood
E-Nerolidol	1.25 (OW)	1.721	1.147	0	Fir, linoleum, pine
Caryophyllene oxide	2.4	0.596	0.213	0.091	Citrus, fruit, herb, must, spice
γ-Dodecalactone	0.00481	0	73.002	0	Apricot, flower, fruit, peach, sweet

**Table 4:** Molecular docking results of key odorants (ROAV > 1) from cultivar F054 with human odorant receptors: shared with and unique to the other two cultivars.

Compounds	Olfactory Receptors	Binding Energy (kcal/mol)	Type of Interactions	Interacting Amino Acids
β-Pinene	OR2W1	−6.973	Hydrophobic Interaction	TYR104, ILE206, VAL207, TYR259
γ-Dodecalactone	OR2W1	−6.422	H-bond, Hydrophobic Interaction	ASN155, PHE73, TYR104, LEU159, VAL207, PHE251, ILE255, TYR259, TYR278
Humulene	OR5AN1	−6.740	Hydrophobic Interaction	TYR74, PHE105, LEU110, THR204, PHE252, VAL259
β-Caryophyllene	OR5AN1	−6.720	Hydrophobic Interaction	LEU110, PHE252, TYR253, VAL259
β-Pinene	OR5AN1	−5.778	Hydrophobic Interaction	PHE105, PHE252, VAL259, TYR279
β-Caryophyllene	OR2AG1	−7.439	Hydrophobic Interaction	LEU105, TYR159, PHE206, LEU207, PHE251, ALA255, TYR259
Humulene	OR2AG1	−6.873	Hydrophobic Interaction	LEU105, TYR159, PHE206, LEU207, PHE251, ALA255, TYR259
γ-Dodecalactone	OR2AG1	−6.008	H-bond, Hydrophobic Interaction	TYR159, LEU207, PHE251, ALA254, TYR259, TYR278
β-Pinene	OR2AG1	−5.875	Hydrophobic Interaction	PHE251, ALA254, TYR259, TYR278
Humulene	OR10G4	−7.343	Hydrophobic Interaction	PHE71, PHE103, ALA206, PHE250, TYR258, V TYR275
β-Caryophyllene	OR10G4	−6.687	Hydrophobic Interaction	PHE103, VAL205, ALA206, PHE250, TYR258, TYR275
β-Pinene	OR10G4	−6.137	Hydrophobic Interaction	PHE71, PHE103, ILE202, TYR275
Humulene	OR5P3	−6.720	Hydrophobic Interaction	VAL104, VAL105, PHE159, ILE207, PHE251, ILE255, TYR278
β-Pinene	OR5P3	−6.457	Hydrophobic Interaction	PHE159, ILE207, TYR259, TYR278
β-Caryophyllene	OR5P3	−6.292	Hydrophobic Interaction	VAL104, VAL105, PHE159, ILE207, ILE255, TYR259, TYR278

## Data Availability

The original contributions presented in this study are included in the article. Further inquiries can be directed to the corresponding authors.
